# Does Peer Rejection Moderate the Associations among Cyberbullying Victimization, Depression, and Anxiety among Adolescents with Autism Spectrum Disorder?

**DOI:** 10.3390/children6030041

**Published:** 2019-03-04

**Authors:** Michelle F. Wright, Sebastian Wachs

**Affiliations:** 1 Department of Psychology, Pennsylvania State University, PA 16802, USA; mfw5215@psu.edu; 2 Faculty of Social Studies, Masaryk University, 60200 Brno, Czech Republic; 3 Department of Educational Studies, University of Potsdam, 14476 Potsdam, Germany

**Keywords:** anxiety, depression, cyberbullying victimization, autism spectrum disorder, peer rejection

## Abstract

While the consequences of cyberbullying victimization have received some attention in the literature, to date, little is known about the multiple types of strains in adolescents’ lives, such as whether cyberbullying victimization and peer rejection increase their vulnerability to depression and anxiety. Even though some research found that adolescents with disabilities show higher risk for cyberbullying victimization, most research has focused on typically developing adolescents. Thus, the present study focused on examining the moderating effect of peer rejection in the relationships between cyberbullying victimization, depression, and anxiety among adolescents with autism spectrum disorder. There were 128 participants (89% male; ages ranging from 11–16 years old) with autism spectrum disorder in the sixth, seventh, or eighth grade at 16 middle schools in the United States. Participants completed questionnaires on cyberbullying victimization, peer rejection, depression, and anxiety. Results revealed that cyberbullying victimization was associated positively with peer rejection, anxiety, and depression among adolescents with autism spectrum disorder. Further, peer rejection was linked positively with depression and anxiety. Peer rejection moderated the positive relationship between cyberbullying victimization and depression, but not anxiety. Implications for prevention programs and future research are discussed.

## 1. Introduction

The digital world is an important developmental context for many adolescents worldwide. Digital technologies offer a constant connection with the world, make vast amounts of information accessible, and are utilized for entertainment. Although there are various benefits associated with digital technology use, there are also negative consequences, including sexual predation, identity theft or misuse, and violence [1]. Cyberbullying victimization is another risk associated with adolescents’ digital technology use. Researchers, educators, parents, and the general public are concerned by cyberbullying victimization because it is associated with an assortment of negative consequences, including depression and anxiety [2,3,4,5,6,7,8]. Peer rejection is also associated with cyberbullying victimization, and it is unclear how peer rejection might worsen the negative outcomes associated with experiencing cyberbullying [9]. In addition, many studies on cyberbullying victimization have utilized samples of typically developing adolescents, with research on adolescents with autism spectrum disorder (ASD) not receiving much attention in the cyberbullying literature.

ASD is a neurodevelopment disorder encompassing autistic disorder, Asperger’s disorder, childhood disintegrative disorder, and pervasive developmental disorder [10]. Because these separate diagnoses were not consistently applied in various clinics and treatment centers across the United States, the Diagnostic and Statistical Manual of Mental Disorders combined these diagnoses into ASD [11]. ASD is characterized by impairments in social communication and interaction, as well as restricted and repetitive behaviors and/or interactions. Adolescents with ASD have trouble with social skills, engage in repetitive behaviors, and are often hypersensitive to environmental stimuli [12]. Such characteristics increase their vulnerability to victimization, as bullies are usually skilled at noticing differences in others [13,14,15]. Many bullies might enjoy aggravating adolescents with ASD because such adolescents might be perceived as easy targets due to environmental hypersensitivity [14].

It is important to focus on adolescents with ASD because they are twice as likely to be victims of bullying, more likely to be rejected by their peers than other adolescents with disabilities, and report greater psychological distress following cyberbullying when compared to typically developing adolescents [16,17,18]. Little attention has been given to whether multiple types of strains in adolescents’ lives, such as cyberbullying victimization and peer rejection, increase their vulnerability to depression and anxiety. To this end, the present study examined whether peer rejection would moderate the associations between cyberbullying victimization, depression, and anxiety among adolescents with ASD. The results might help to deepen our knowledge on a vulnerable group particularly susceptible to cyberbullying victimization and the possible negative consequences of cyberbullying. In addition, this study can provide information for prevention and intervention efforts to tackle cyberbullying among adolescents. Furthermore, findings from this research could help inform the development of prevention and intervention programs that are more inclusive of adolescents with ASD.

### 1.1. Cyberbullying Victimization among Adolescents with Disabilities

Defined as purposefully being repeatedly targeted by hostile, embarrassing, and/or intimidating behaviors via the internet and other digital technologies, cyberbullying victimization might also involve an imbalance of power between the perpetrator and victim [19,20,21]. Cyberbullying victimization involves receiving abusive messages, being the target of identity theft, harassment, physical threats, social exclusion, and humiliation [22,23,24]. Such victimization might also involve having embarrassing or explicit pictures or videos distributed of oneself, and being targeted by flaming, trolling, and hacking [25,26,27]. There is a relationship between being bullied offline and being cyberbullied [28]. Cyberbullying victimization is linked to a variety of negative outcomes, including depression and anxiety [2,3,4,5,28].

Digital technology use is a risk factor associated with experiencing and perpetrating cyberbullying [24,27,29,30]. Adolescents with disabilities also utilize digital technologies, which could increase their risk of cyberbullying victimization [31]. Kuo et al. [32] found that adolescents with ASD utilized home computers at least five hours a day, and that they preferred using non-social media. Little attention has been given to rates of cyberbullying victimization among adolescents with ASD. In one study with adolescents who were diagnosed with high functioning autism, Kowalski and Fedina [17] found that these adolescents reported experiencing cyberbullying victimization and that they had greater levels of physical and psychological health problems when compared to typically-developing adolescents who were uninvolved with cyberbullying. Other research has found that adolescents with disabilities were more likely to report distressing cyberbullying victimization when compared to adolescents without disabilities [18]. Wright [33] also found that cyberbullying victimization and face-to-face bullying victimization were associated among adolescents with ASD, and that cyberbullying victimization was also related to depression and anxiety among these adolescents. Taken together, these studies suggest that adolescents with special needs might be particularly vulnerable to cyberbullying victimization and the associated negative outcomes.

### 1.2. Peer Rejection

Many adolescents are concerned with their social reputation in their peer group [34,35]. Adolescents who are rejected by their peers might be repeatedly exposed to negative attitudes over a long period of time [36,37]. Some rejected adolescents receive negative treatment from their peers because they might be immature, socially unskilled/awkward, timid/shy, withdrawn, or lack positive social traits, such as kindness and honesty [38,39,40]. Peer rejection is linked to stable patterns of peer victimization during early, middle and late childhood, as well as during adolescence [41,42,43,44,45]. Peer rejection is hypothesized to be a determinant of peer victimization as rejection increases children’s and adolescents’ vulnerability to victimization [46,47]. Oftentimes rejected children and adolescents are isolated from the peer group, making them vulnerable to peer victimization because their peers are unable to protect them from being bullied or show disapproval toward the attacker. Because they lack protection from their peers, rejected children and adolescents are often victimized.

Social standing among peers, including peer rejection, is influenced by adolescents’ disability status. In their research, Pijl, Frostad, and Flem [48] compared the social status of adolescents with various disability types (i.e., students with behavioral challenges, mild to severe learning disabilities, and communication problems) in middle school and high school. They concluded that adolescents with behavioral or communication problems were at a greater risk for social isolation when compared to adolescents with sensory or motor disabilities. Similar patterns were found by Symes and Humphrey [49], who found that adolescents with autism spectrum disorder had higher levels of peer rejection and lower levels of peer acceptance when compared to their typically developing peers. Such findings were attributed to the general negative attitudes concerning peers with autism spectrum disorder among general education students [50,51].

Research has also revealed a positive correlation between peer rejection and cyber victimization among adolescents [9]. Such a finding is expected as adolescents’ nondigital identities usually overlap with their online identities, indicating that peer victimization might continue into the online environment for rejected adolescents [52].

Peer rejection is a positive predictor of antisocial behaviors, depression, social anxiety, poor academic performance, low school engagement, and sexual risk behaviors during adolescence [53,54,55,56,57]. Some research has also focused on the moderating effect of peer rejection in the association between cyberbullying victimization and perpetration. Wright and Li [9] found that higher levels of peer rejection increased the positive association between cyberbullying victimization and perpetration. Other research has revealed that peer rejection strengthened the positive relationship between aggression and externalizing behaviors among adolescent girls [57]. Sandstrom and Schanberg [58] examined the moderating effect of peer rejection in the relationship between pain and depression among children with juvenile rheumatic disease. Their findings revealed that children with high levels of pain and more peer rejection reported greater depressive symptoms. These studies suggest that peer rejection is an important moderating factor because it has the potential to exacerbate the strain associated with cyberbullying victimization and the negative consequences, such as depression and anxiety.

### 1.3. The Present Study

Considering that cyberbullying victimization is associated positively with peer rejection, depression, and anxiety, and that peer rejection is related positively to depression and anxiety, it might be likely that higher levels of peer rejection could strengthen the associations between cyberbullying victimization, depression, and anxiety among adolescents with autism spectrum disorder. To this end, the present study investigated the moderating effect of peer rejection in the relationships between cyberbullying victimization, depression, and anxiety. The following hypotheses were created to guide this research:

**Hypothesis** **1** **(H1).** *Cyberbullying victimization will relate positively to peer rejection, anxiety, and depression among adolescents with autism spectrum disorder.*

**Hypothesis** **2** **(H2).** *High peer rejection among adolescents with autism spectrum disorder will be associated positively with depression and anxiety.*

**Hypothesis** **3** **(H3).** *High levels of peer rejection will strengthen the positive associations among cyberbullying victimization, depression, and anxiety.*

## 2. Methods

### 2.1. Participants

There were 128 participants (89% male; ages ranging from 11–16 years old; M age = 13.76; SD = 0.86) with autism spectrum disorder in the sixth, seventh, or eighth grade at 16 middle schools. The middle schools were located in the suburbs of a large Midwestern city in the United States. Participants were diagnosed with one of the following autism spectrum disorders: autistic disorder, Asperger syndrome, or pervasive developmental disorder. The middle schools were in middle-class neighborhoods and about 32% of students at the schools received free or reduced cost lunch. Participants primarily identified as white (86%), followed by Asian (10%), Black/African American (3%), and Latino/a (1%). No income data was collected from participants’ families.

### 2.2. Procedures

The research was approved by the DePaul University ethic’s board (#MW080210PSY). American Psychological Association ethical standards were followed when conducting this study. This research is part of a larger project on adolescents’ peer relationships, digital communications, and online and offline behaviors. From a list of over 150 public schools, 30 middle schools were randomly selected. Emails were sent to school principals, which described the purpose of the study, what the adolescents would be expected to do, and how long the study would take. There were 16 school principals who expressed interest in the study. District approval was also obtained from 12 of the 16 middle schools; the other four schools required principal-level approval. Meetings were set up between the school principals, teachers, and the principal investigator. The purpose of the meeting was to describe the research, who would participate, and how long the study would take. As part of the larger project, the principal investigator met with school psychologists to help identify adolescents with the diagnosis of an autism spectrum disorder and who were able to read questionnaires without the help of a paraprofessional. Data was not collected on the specific number of adolescents with different ASD diagnoses. The diagnosis of ASD was not performed by the researchers and instead was provided by the school psychologists. With the help of the school psychologists, 201 eligible adolescents were identified. Announcements were made to these adolescents, with a graduate assistant who had specialized training in autism spectrum disorder present. In the announcement, the purpose of the study was described, along with what adolescents would be expected to do and how long the study would take. A letter and parental permission slip were distributed to adolescents. Of the 201 parental permission slips sent home, 138 parental permission slips were returned, 45 were returned without permissions, and the rest were never returned. Data were collected in January and February of 2016.

Before data collection, adolescents provided their assent. Five adolescents did not provide their assent, and were sent back to their classroom. Another five adolescents were not present during data collection, and thus were not included in the study. Adolescents with autism spectrum disorder completed questionnaires on demographic information (e.g., age, gender, ethnicity), traditional face-to-face bullying victimization, cyberbullying victimization, depression, anxiety, and peer rejection. Research assistants trained in special education were present to answer any questions that adolescents had during data collection.

### 2.3. Measures

#### 2.3.1. Traditional Face-To-Face Bullying Victimization

There were 12 items included on this questionnaire, which assessed how often adolescents experienced traditional face-to-face bullying victimization. The adolescents answered questions on a scale of 1 (*not at all*) to 5 (*all of the time*). The reference period was “during the current school year.” A sample item included: “A peer called me insulting names” [59]. Cronbach’s alpha was 0.83.

#### 2.3.2. Cyberbullying Victimization

Adolescents reported how often they experienced victimization online or via text messages [9]. The reference period was “during the current school year.” The adolescents answered eight items on a scale of 1 (*not at all*) to 5 (*all of the time*). A sample item included: “A peer insulted me by calling me mean and humiliating names online or through text messages.” Cronbach’s alpha was 0.86.

#### 2.3.3. Depression

The Center for Epidemiological Studies Depression Scale was used to assess adolescents’ depressive symptoms [60]. They rated 20 items on a scale of 0 (*rarely or none of the time*) to 3 (*most or all of the time*) according to how they have felt in the past week. A sample item included: “I did not feel like eating, my appetite was poor.” Cronbach’s alpha was 0.86.

#### 2.3.4. Anxiety

The Multidimensional Anxiety Scale for Children was used to assess adolescents’ anxiety symptoms in the past week [61]. Adolescents rated 39 items on a scale of 0 (*never true about me*) to 3 (*often true about me*). A sample item included: “I get scared when my parents go away.” Cronbach’s alpha was 0.87.

#### 2.3.5. Peer Rejection

Peer rejection scores were obtained from the larger project (*n* = 1189 adolescents between 11 and 15 years old who did not have an ASD diagnosis), with scores only included for adolescents in this smaller project. Adolescents from the larger project, as well as adolescents in this smaller project, were asked to nominate as many peers as they wanted within their grade who fit the description of “Peers whom you like the least” [45,62,63,64]. Adolescents utilized a sheet of paper with the names of students in their grade and associated identification codes (IDs) and they recorded only the IDs on the questionnaire. All nominations received were tallied and then standardized within each grade. Higher scores indicated that the peer was not well-liked or was rejected by their peers.

### 2.4. Analytical Plan

Two multiple hierarchical regression analyses were conducted for the two adjustment outcomes of depression and anxiety using Statistical Package for the Social Sciences (SPSS) Version 25 (Armonk, NY, United States). Predictor variables included cyberbullying victimization and peer rejection. Gender and traditional face-to-face bullying victimization were included as covariates in Block 1. Block 2 included cyberbullying victimization. Block 3 included peer rejection. Block 4 included an interaction between cyberbullying victimization and peer rejection. Continuous predictor variables were centered. The Interaction program was used to provide graphical representation of simple slopes of the regression lines, unstandardized betas, and significance levels [65].

## 3. Results

Means, standard deviations, and correlations were calculated among all variables in the study (Table 1). Cyberbullying victimization was related positively to traditional face-to-face bullying victimization, peer rejection, depression, and anxiety. Peer rejection was associated positively with traditional face-to-face bullying victimization, depression, and anxiety. Traditional face-to-face bullying victimization was related positively to depression and anxiety. There were small effect sizes for the correlations.

**Figure 1:**
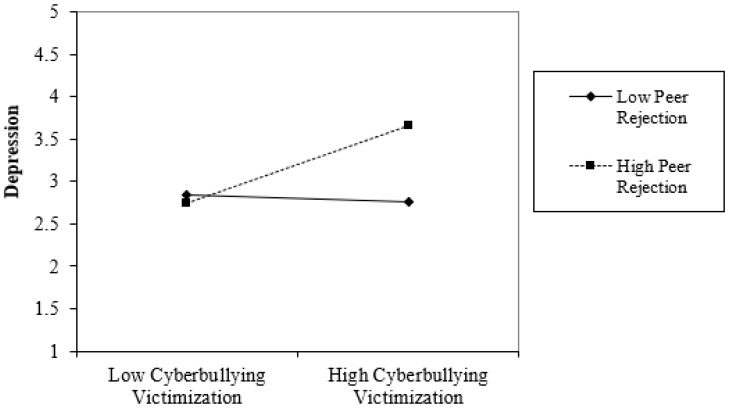
Graphical representation of the interaction between cyberbullying victimization and peer rejection for depression.

For the multiple regression analyses, only the highest significant block will be reported. Gender was unrelated to depression or anxiety (see Table 2). Face-to-face traditional bullying victimization was related positively to depression (β = 0.16, *p* < 0.05) and anxiety (β = 0.17, *p* < 0.05). Cyberbullying victimization was associated positively with depression (β = 0.21, *p* < 0.05) and anxiety (β = 0.20, *p* < 0.05). High peer rejection was correlated positively with depression (β = 0.26, *p* < 0.05) and anxiety (β = 0.26, *p* < 0.01). The two-way interaction between cyberbullying victimization and peer rejection was significant for depression (β = 0.25, *p* < 0.01) but not for anxiety (β = 0.10, p = n.s.). High levels of peer rejection increased the positive relationship between cyberbullying victimization and depression (β = 0.20, SE = 0.07, *p* < 0.001, +1 SD) (Figure 1). The slopes for low levels of peer rejection and mean levels were not significant.

## 4. Discussion

Cyberbullying victimization and peer rejection are two sources of strain experienced by adolescents with ASD. Both types of strain might exhaust these adolescents’ effective coping strategies, leading to negative outcomes such as depression and anxiety. One direction missing in the literature on the negative outcomes of cyberbullying victimization is the joint contribution of multiple strains. Investigations aimed at understanding how cyberbullying victimization and peer rejection could worsen depression and anxiety are important, as this information can be used for intervention programs designed to teach effective coping strategies in an effort to reduce negative emotions elicited from experiencing multiple strains at once. To address the gap in the literature on the effects of experiencing multiple types of strain, the present study examined whether cyberbullying victimization might be higher for adolescents who experienced peer rejection, as well as how peer rejection might moderate the associations among cyberbullying victimization, depression, and anxiety. Our study provides an early effort to examine the joint contributions of cyberbullying victimization and peer rejection on depression and anxiety among adolescents with ASD.

Consistent with our first hypothesis, we found that cyberbullying victimization was associated positively with peer rejection, anxiety, and depression among adolescents with ASD. The finding regarding the positive correlation between cyberbullying victimization and peer rejection is consistent with the literature [9]. Furthermore, our findings expand the literature on the association between traditional face-to-face bullying victimization and peer rejection to the cyber context, suggesting that similar mechanisms linking the experience of victimization to peer rejection might also occur in cyberspace [41,42,43,44]. Behavioral experiences are often similar in the offline world as they are in the online world among adolescents, potentially increasing the risk of cyberbullying victimization among adolescents with ASD who are also likely to be victimized offline [52]. Our findings might also further suggest that rejected adolescents’ experiences follow them into the online world. In particular, adolescents’ characteristics that might contribute to their peer rejection among their peers in the offline world are consistent and potentially contribute to their online experiences. Such a proposal is logical as adolescents often interact with peers their own age and from their offline worlds [66]. Finding that cyberbullying victimization is related positively to anxiety and depression is also consistent with the literature [2,3,4,5,7,8,28].

We found that high peer rejection was linked positively with depression and anxiety among adolescents with ASD, providing support for our second hypothesis. This finding is consistent with the literature [57]. Concerns about one’s social standing among peers is heightened during early adolescence [34,35,67]. Therefore, early adolescents might feel especially distressed over their low status and feel that their low status is particularly salient among their peer group. Rejected adolescents might repeatedly experience negative attitudes from their peers, potentially increasing their vulnerability to depression and anxiety [36,37]. Ruminating over experiences is a characteristic of ASD, and their repeated exposure to negative attitudes and rejection might be experiences that these adolescents continuously relive [68]. Furthermore, research also indicates that adolescents with ASD are aware of their status and emphasize peer approval similar to their typically developing peers [69]. Considering that adolescents with ASD might have higher levels of peer rejection and lower levels of peer acceptance, these adolescents might also be at a greater risk of depression and anxiety [49].

Our findings are best understood through the significant interaction between cyberbullying victimization and peer rejection when predicting depression. We found that the positive relationship between cyberbullying victimization and depression was greater when peer rejection was high, providing some support for our third hypothesis. Little attention has been given to the moderation of peer rejection. In the most similar available research, Wright and Li [9] found that high peer rejection increased the positive association between cyberbullying victimization and perpetration. Other research has revealed that peer rejection increases the association of externalizing problems and depression to aggression and physical pain [57,58]. Considering the conclusions from each of these studies, it is clear that joint forms of strain (i.e., cyberbullying victimization and peer rejection, aggression and peer rejection, physical pain and aggression) increase adolescents’ experience of negative outcomes, such as cyberbullying perpetration, externalizing problems, and depression. Therefore, our findings expand this literature by indicating that multiple forms of strain, such as cyberbullying victimization and peer rejection, are associated with depressive symptoms among adolescents with ASD. It is likely that the strain of cyberbullying victimization and peer rejection weakened these adolescents’ ability to cope, resulting in negative emotions that potentially lead to depression.

Peer rejection did not moderate the association between cyberbullying victimization and anxiety among adolescents with ASD, as proposed in our third hypothesis. A lack of research makes it difficult to explain this finding. Wright [33] did not find support that high parental mediation moderated the relationship between cyberbullying victimization and anxiety among adolescents with autism spectrum disorder, although she found support for moderation in samples of typically developing adolescents [6,33]. Furthermore, much of the research linking peer rejection to anxiety among adolescents with ASD focuses on social anxiety [57]. The measure of anxiety in this study was a generalized assessment. More research should be conducted to better understand the role of peer rejection in cyberbullying victimization and social anxiety among adolescents with ASD.

Even though the present study revealed valuable information on the joint contributions of cyberbullying victimization and peer rejection on depression and anxiety among adolescents with ASD, a number of limitations require mention. First, one limitation of this study is the cross-sectional nature of the data. Although it is theoretically sound to propose that the cyberbullying victimization leads to higher levels of depression and anxiety there is also some empirical evidence for a reciprocal relationship between cyberbullying victimization, depression, and anxiety [7,8]. To draw final conclusions about temporal ordering and causality, either a longitudinal or an experimental design is needed. Second, the sample consisted of adolescents attending the sixth, seventh, or eighth grade and most of the participants were male. Therefore, the sample cannot be considered representative, which limits findings to a specific population. This research might also benefit from comparing adolescents with ASD to adolescents with other developmental disabilities or those who are typically developing. Future studies should include representative samples to increase the generalizability of the present study’s findings. Third, cyberbullying victimization, depression, and anxiety measures were self-reported. Follow-up studies could combine peer-reported and self-reported cyberbullying victimization and parent or teacher reports of depressive and anxiety symptoms. Third, in the present study only adolescents with ASD were included. Future studies should consider a wider range of adolescents with disabilities, such as deafness, learning disabilities, and language impairment, and compare these groups regarding their risk for cyberbullying victimization and potential negative outcomes. Finally, in the present study a global approach for measuring anxiety was applied. Future research could test whether peer rejection moderates the association between cyberbullying victimization and specific forms of anxiety (e.g., social anxiety disorder). This research could also consider specific types of cyberbullying victimization (i.e., denigration, harassment, exclusion, outing). The diagnosis of ASD was provided by the school, making it difficult to determine if diagnoses were similarly applied across adolescents included in this study. In addition, depression scores were within normal range for adolescents, and therefore follow-up research might benefit from including adolescents with higher scores on depression to determine if the findings of the present research apply to other samples of adolescents.

## 5. Conclusions

The present study investigated the joint effects of cyberbullying victimization and peer rejection on depression and anxiety among adolescents with ASD. Adolescents with ASD experience peer victimization, are rejected by their peers, and potentially ruminate over their low social standing while also being concerned with their status. These experiences and characteristics might increase their vulnerability to depression and anxiety. This study was one of the first to document the finding that high peer rejection strengthened the positive association between cyberbullying victimization and depression (but not anxiety) among adolescents with ASD. Such findings support the need for additional research investigating the risk factors associated with increasing the negative effects of cyberbullying victimization among adolescents with ASD. Multifaceted solutions are required to increase parents’ and educators’ awareness of the dangers associated with experiencing multiple sources of strain through cyberbullying victimization and peer rejection. Solutions to reduce negative outcomes associated with cyberbullying victimization among adolescents are warranted, such as teaching them effective coping strategies or other ways to reduce negative emotions and stress. It is important that these solutions apply to adolescents with ASD as well.

## Figures and Tables

**Table 1:** Correlations among all variables.

	1	2	3	4	5
1. Traditional Face-to-face Bullying Victimization	---				
2. Cyberbullying Victimization	0.51 ***	---			
3. Peer Rejection	0.29 ***	0.31 ***	---		
4. Depression	0.38 ***	0.30 ***	0.28 **	---	
5. Anxiety	0.31 ***	0.26 **	0.23 *	0.41 ***	---
*M* (*SD*) for Full Sample	3.26 (1.06)	2.97 (0.97)	0.16 (0.36)	1.98 (0.68)	1.81 (0.79)
*M (SD)* for Boys	3.29 (1.10)	2.99 (1.00)	0.18 (0.41)	1.97 (0.67)	1.80 (0.79)
*M (SD)* for Girls	3.23 (1.01)	2.95 (0.93)	0.14 (0.31)	1.99 (0.68)	1.83 (0.80)

* *p* < 0.05. ** *p* < 0.01. *** *p* < 0.001.

**Table 2:** Cyberbullying victimization, peer rejection, depression, and anxiety.

	Depression			Anxiety		
	*β*	*R2*	ΔR2	*β*	*R2*	ΔR2
Block 1		0.36	0.36 *		0.38	0.38 *
Gender	0.02			0.03		
F2F Vic	0.21*			0.23*		
Block 2		0.43	0.07 ***		0.44	0.06 ***
Gender	0.01			0.01		
F2F Vic	0.17*			0.21*		
CVic	0.30***			0.30***		
Block 3		0.50	0.07 ***		0.48	0.04 ***
Gender	0.03			0.02		
F2F Vic	0.17*			0.17*		
CVic	0.21*			0.20*		
PR	0.26**			0.26**		
Block 4		0.53	0.03 **		0.49	0.01
Gender	0.02			0.01		
F2F Vic	0.16*			0.20		
CVic	0.21*			0.19		
PR	0.20*			0.06		
CVic x PR	0.25**			0.10		

F2F Vic—Traditional Face-to-face Bullying Victimization; CVic—Cyberbullying Victimization; PR—Peer Rejection. * *p* < 0.05. ** *p* < 0.01. *** *p* < 0.001.
